# The Chlorination of Water

**Published:** 1921-10

**Authors:** 


### CHLORINATION OF WATER.

### CIRCULAR OF THE MINISTRY OF HEALTH.

The rapid sterilisation of drinking-water by means of chlorine is described in an appendix to Circular 241, issued by the Ministry of Health on September 15 to local authorities

#### General Principles of Chlorination.

Chlorine is most readily available in the form of chloride of lime (bleaching powder), which can readily be obtained commercially. This, if freshly made, should contain 33 per cent. of free or active chlorine. It loses its efficacy on keeping, especially during hot weather; but this loss may be retarded by the addition of 25 per cent. of quicklime. Samples of the bleaching powder used should be examined by a chemist to see that the proper amount of chlorine is present. Any deficiency in strength may be counteracted by an increased dose.

In order to be sure that all infective organisms are killed, the chloride of lime must be added to the water in such quantity that free chlorine will be available to the extent of at least one part per million of water. This will be effected by the preparation of a solution of chloride of lime consisting of 1 ounce of chloride of lime to 1 quart of water. This will suffice to treat 2,000 gallons of a water which does not contain an excessive amount of mineral or organic matter.

If the water is very impure, discoloured, or contains much suspended matter, it should, if circumstances permit, first be clarified by the addition of from 2 to 8 grains per gallon of alum and an equal proportion of powdered whiting, and allowed to stand in a tank, from which it should be decanted to another tank for the addition of the chloride of lime solution.

#### Removal of the Taste of Chlorine.

After the addition of the chloride of lime, the water should, *if practicable*, be allowed to stand in an open tank for four hours before consumption. If this can be effected there will usually be no complaint of taste of chlorine in the water. If this cannot be effected the taste may often be removed by the addition of a small quantity of permanganate, sufficient to produce a very faint pink colour fading away in about five to fifteen minutes; or, so far as the chlorine taste is concerned, by the addition of a solution of sodium thiosulphate (the hyposulphite of soda used in photography) in small quantities. The quantities of these preparations which are necessary to remove the taste can be quickly determined by practical tests. Wherever possible, in the case of a water supply which is filtered, the chlorine solution should be added after filtration.

#### Adaptation of Method to Quantity.

There are various ways of adding the chlorine solution to the water. If it is a small supply, such as that from a cottage well, dip well, small spring, or land drain, the water will have to be pumped into some receptacle such as a tub, barrel, or tank-cart, and the chlorine solution added by hand. Water should be allowed to stand for four hours in the open, and then be distributed to consumers.

If it is a larger supply, corresponding methods, involving the adding of measured quantities of the chlorine solution by hand, will probably have to be adopted in the first instance, because it takes time to provide or arrange apparatus.

If the treatment has to be continued for any length of time, however, an improvement on this method is to provide a small tank to hold the chlorine solution, which is fed from a larger tank, and kept filled to a certain level by means of a ball tap or other similar contrivance. In this way, a constant level, and therefore constant pressure, is maintained in the small tank, which assists in regulating the flow. If this is fitted with a fine glass tube, having a controlling tap and a pressure gauge, the solution may be allowed to drip at such a rate as to pass the required amount per hour into the water, the rate of discharge of which into the reservoir is known. The tap should be so arranged as to drip over the point of discharge of the water into the reservoir. When the reservoir is filled, the chlorinated water should be allowed to stand for four hours before consumption, if practicable.

When the chlorine cannot be added to the water in the reservoir the solution may be injected into the rising or other main by means of a suitable contrivance, such as a pump driven by a turbine from the rising main, the amount of solution injected being proportionate to the rapidity of flow of water in the main, a definite relation being established between the two.

The tank containing the chlorine solution should preferably be of glass, in order that any failure in the supply may be detected at a glance.

#### Difficulties and Safeguards

The continued use of chloride of lime, except where employed on a very large scale, and under skilled supervision, may give rise to difficulties in that it requires labour and constant attention, and it will probably be found that some other method of adding the chlorine will in the long run be preferable if the treatment has to be continued for an indefinite period. Various liquid preparations containing chlorine in known proportions may be employed, such as "Chloros," which contains from 10 to 15 per cent. of available or free chlorine and is prepared by the United Alkali Co., Liverpool. The liquefied gas can also be employed, and there are various forms of apparatus for effecting this automatically, also for using the dry chlorine gas or a solution of this in water.

Any mains, pipes, cisterns, or tanks which have contained polluted water should be emptied and disinfected. It will frequently be found, after the water mains, pipes, and cisterns have been disinfected by a strong solution, say, five parts per million, and after the use of chlorine, in the proportion of one part per million, for some days in the water, that the amount of chlorine may be reduced to one part in three millions, and risk of "taste" will then be reduced. Whether this can be done will depend on the course which the outbreak of disease takes, and on the results of analysis, bacteriological and chemical, of the water before and after treatment with the chlorine.

